# Spontaneous colonic perforation in a pediatric patient with acute febrile illness: a case report

**DOI:** 10.1093/jscr/rjaf1026

**Published:** 2026-01-08

**Authors:** Abhiraj Yadav, Neha Arutla, Shreya Muddana, Hannah S Jenifer, Malavath Sharath, Noorudin Ansari

**Affiliations:** Manipal College of Medical Sciences, Emergency Medicine, Fulbari, Pokhara, 33700, Nepal; Mamata Academy of Medical sciences, General Surgery, Hyderabad, 500118, India; Mamata Academy of Medical sciences, General Surgery, Hyderabad, 500118, India; Mamata Academy of Medical sciences, General Surgery, Hyderabad, 500118, India; Mamata Academy of Medical sciences, General Surgery, Hyderabad, 500118, India; Department of General Surgery, Mamata Academy of Medical sciences, Fulbari, Hyderabad 500118, India

**Keywords:** spontaneous colonic perforation, pediatric acute abdomen, ascending colon perforation, right hemicolectomy, idiopathic bowel perforation

## Abstract

Spontaneous colonic perforation is a rare but life-threatening surgical emergency, particularly uncommon in children without predisposing conditions. We report a 4-year-old male who presented with acute febrile illness, suprapubic abdominal pain, and dysuria, initially managed as cystitis. Despite treatment, he developed peritonitis; imaging revealed free subdiaphragmatic air. Exploratory laparotomy showed an ischemic ascending colon with a solitary perforation, necessitating right hemicolectomy with ileocolic anastomosis. Histopathology demonstrated nonspecific ileitis, typhlitis, and colitis complicated by cecal perforation, with no evidence of typhoid, tuberculosis, or inflammatory bowel disease. Postoperative recovery was uneventful following broad-spectrum antibiotics. This case highlights the diagnostic challenge of spontaneous colonic perforation in children, where non-specific presentations may mimic urinary tract or infectious illnesses. Early recognition and timely surgical intervention are crucial to reduce morbidity and mortality. To our knowledge, this represents one of the few pediatric reports of spontaneous ascending colon perforation in the absence of identifiable etiology.

## Introduction

Spontaneous perforation of the colon is defined as a sudden perforation of an otherwise healthy colon in the absence of any predisposing disease or injury. The earliest case was described by Brodie in 1827, who reported a woman with spontaneous rectal rupture, and since then, fewer than 100 cases have been documented in the literature [1]. More than 60% of colonic perforations occur in the sigmoid or at the recto-sigmoid junction, most often along the anti-mesenteric border, making spontaneous cecal perforation a rare entity [2]. We report such a case that occurred spontaneously in a 4-year-old boy in a setting of acute febrile illness.

## Case presentation

A 4-year-old male child, weighing 18 kg, was brought to the pediatric outpatient department with a 1-day history of fever and abdominal pain. He had been well until the day prior, when he developed a high-grade, intermittent fever associated with chills and rigors. The fever was partially relieved by antipyretics, and the child remained active during afebrile periods. There was no associated rash.

The abdominal pain was localized to the suprapubic region, dull and aching in nature, and was relieved following micturition. It was accompanied by dysuria, characterized by burning sensation during urination, along with reduced frequency and quantity of urine. There was no hematuria, dribbling, or straining. A concurrent non-productive cough was reported, but there was no sore throat, vomiting, loose stools, or recent travel. Appetite was reduced, though no weight loss was noted.

The child’s past history was notable for hospitalization with chickenpox 6 months prior. He was fully immunized for age, developmentally normal, on a mixed diet, and had no drug or food allergies. Family history was unremarkable for urinary or renal disease.

On examination, suprapubic and periumbilical tenderness was elicited, with no distension or rigidity. Bowel sounds were normal. Abdominal ultrasonography revealed floating internal echoes in the urinary bladder, suggestive of cystitis. The child was admitted and started on intravenous fluids, antibiotics, analgesics, antispasmodics, and antipyretics. Despite treatment, he continued to have high-grade fever, abdominal pain, bilious vomiting, reduced urinary frequency, and dark-colored urine. Repeat ultrasonography demonstrated a relatively prominent ascending colon with mild wall thickening. On examination, diffuse tenderness with guarding and rigidity was noted, and a clinical diagnosis of peritonitis was made.

**Figure 1:**
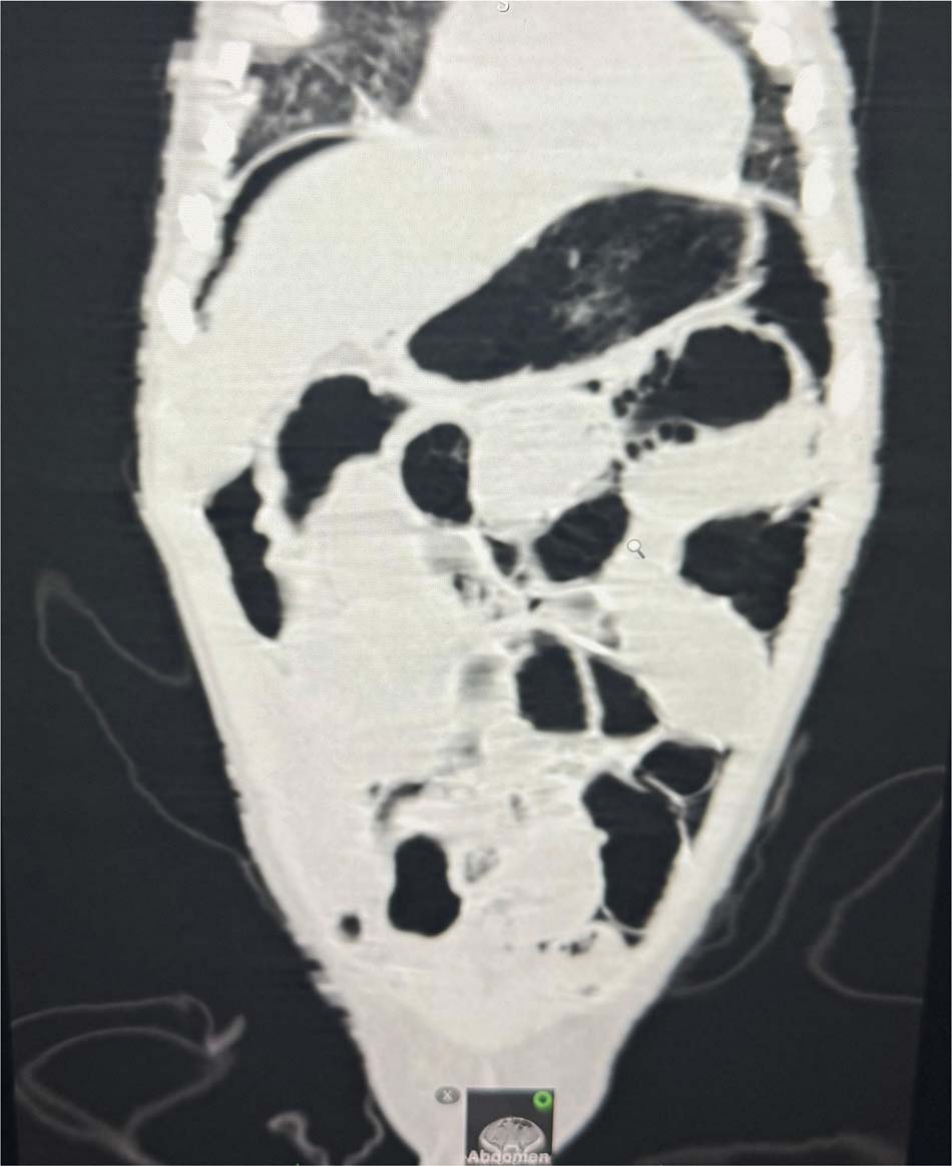
CT abdomen revealed free air under the diaphragm, indicating hollow viscus perforation.

**Figure 2:**
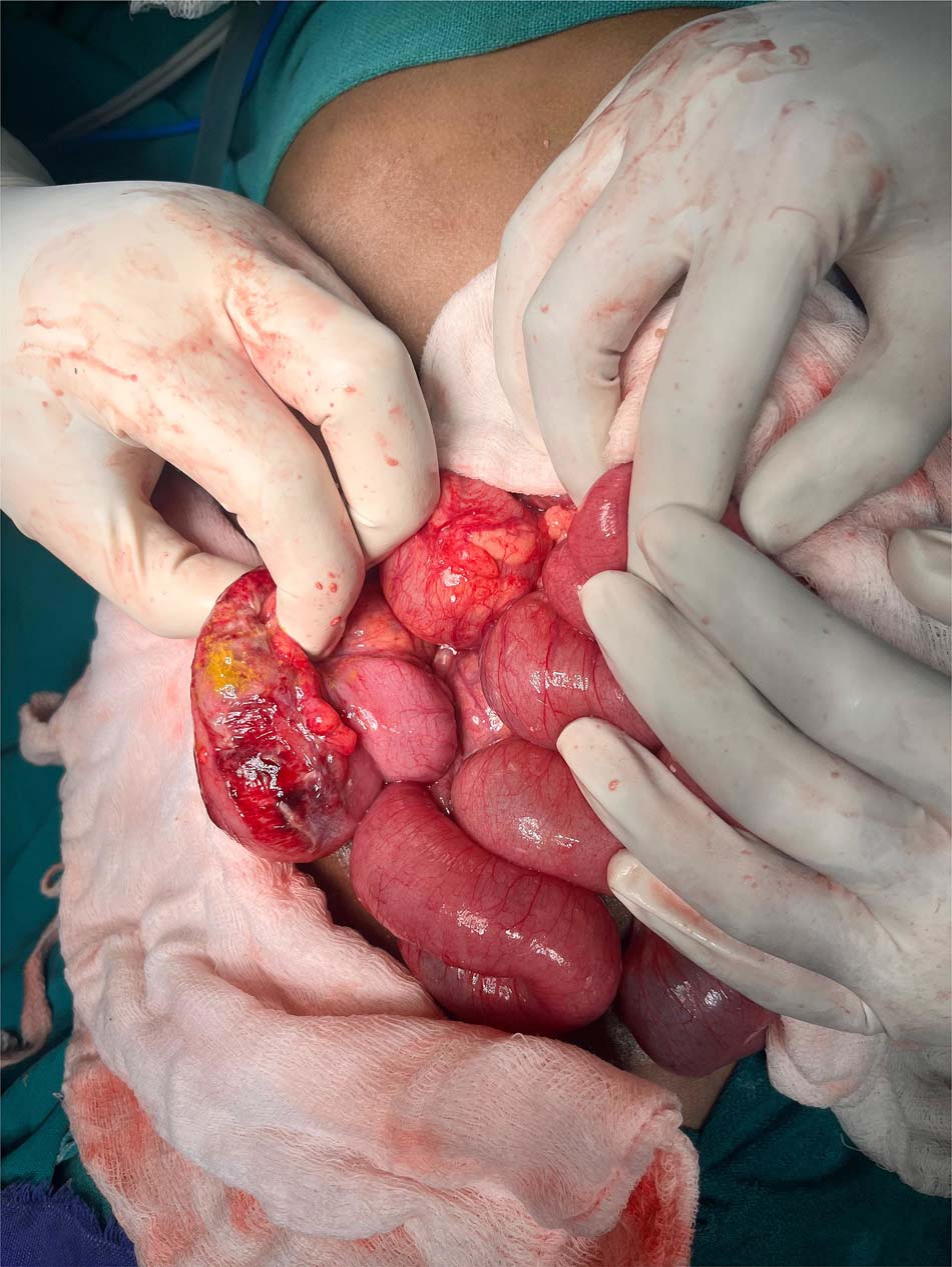
Intraoperative findings show the ileo-cecal region, which is inflamed, edematous, and ischemic, with a solitary cecal perforation and leakage of fecal matter.

**Figure 3:**
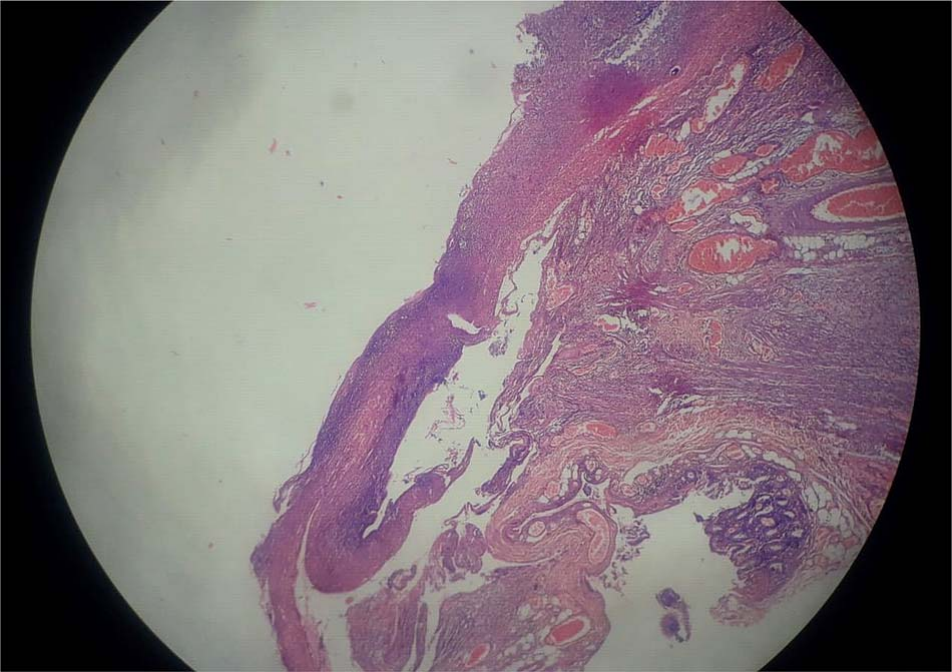
Histopathology revealed acute on chronic non-specific ileitis with typhlitis showing transmural mixed inflammatory infiltrates and reactive lymphoid hyperplasia without granulomas.

An abdominal X-ray, erect, and computed tomography (CT) abdomen revealed free subdiaphragmatic air, suggestive of hollow viscus perforation (Fig. 1). Exploratory laparotomy was performed, which revealed an inflamed, edematous, ischemic ascending colon with a solitary perforation leaking fecal matter (Fig. 2). Enlarged mesenteric lymph nodes were present, while the appendix and rest of the bowel appeared normal. A right hemicolectomy with side-to-side ileocolic anastomosis was performed. The resected specimen, comprising ileum, cecum, appendix, and ascending colon, on histopathology revealed acute on chronic nonspecific ileitis with typhlitis and colitis, complicated by cecal perforation (Fig. 3).

Postoperatively, the child was on injection piperacillin and tazobactam; he remained afebrile for 2 days and later developed fever for 3 days without any wound infection. Complete blood counts, urine examination, dengue serology, and blood cultures were all negative. Intravenous meropenem was started, after which he became afebrile. His recovery was uneventful, and at follow-up, he remained asymptomatic with normal bowel habits.

## Discussion

Spontaneous colonic perforation is extremely rare, but a life-threatening surgical emergency, and pediatric cases are usually secondary to typhoid fever, tuberculosis, Hirschsprung’s disease, necrotizing enterocolitis, trauma, steroid or NSAID use, or inflammatory bowel disease [3].When no such underlying cause is found, the event is considered idiopathic and is exceedingly uncommon in the pediatric population.

Our patient, initially managed for presumed urinary tract infection, progressed to peritonitis, with surgery revealing an isolated ascending colon perforation and histology confirming non-specific inflammation excluding typhoid, tuberculosis, and other infectious causes. This is unusual, as most reported pediatric colonic perforations occur in the rectosigmoid junction, where vascular supply is vulnerable [4].

The exact mechanism of spontaneous colonic perforation is unclear, but proposed explanations include vascular compromise causing focal ischemia, mucosal inflammation, and increased intraluminal pressure that weakens the bowel wall. Clinicopathologic studies in children have demonstrated ischemic necrosis and acute inflammation at perforation sites, supporting these mechanisms [5]. In our case, the presence of ischemic bowel and mesenteric lymphadenopathy at surgery points toward an inflammatory or vascular insult, but the occurrence in a previously healthy febrile child is scarcely reported.

Early recognition and prompt surgical management are essential as delays lead to sepsis and high mortality. In our case, right hemicolectomy with ileocolic anastomosis was performed due to widespread ischemia, consistent with prior pediatric reports recommending resection when bowel margins are unhealthy [6].

This case emphasizes two important implications. First, persistent fever with abdominal pain in children should prompt evaluation for intra-abdominal pathology, even when initial findings suggest conditions such as urinary tract infection. Second, although rare, spontaneous colonic perforation must be considered in the differential diagnosis of pediatric acute abdomen.

Our report contributes to this scarce body of evidence and, to our knowledge, is among the very few documenting spontaneous ascending colon perforation in a febrile child without an identifiable underlying cause.

## Conclusion

Spontaneous colonic perforation in children is exceedingly rare and often life-threatening. This case of isolated ascending colon perforation in a previously healthy febrile child underscores the importance of considering intra-abdominal pathology when fever and abdominal pain persist, even when initial investigations suggest alternative diagnoses. Early recognition and timely surgical intervention remain critical to improving outcomes.

## References

[1] Al-Balas H, Al-Balas M, Al-Wiswasy M. Idiopathic spontaneous cecal perforation: a rare pathology with high mortality. Ann Med Surg 2020;2012:518–21. 10.1016/j.amsu.2020.11.047 PMC7695924 33294185

[2] Makki AM, Hejazi S, Zaidi NH, et al. Spontaneous perforation of colon: a case report and review of literature. Case Reports in Clinical Medicine 2014;3:392–7. 10.4236/crcm.2014.37087

[3] Saraç M, Bakal Ü, Aydın M, et al. Neonatal gastrointestinal perforations: the 10-year experience of a reference hospital. Indian J Surg 2017;79:431–6. 10.1007/s12262-016-1565-z 29089704 PMC5653578

[4] Solomon N, Habte T, Alemu S, et al. Spontaneous rectosigmoid perforation at the watershed area of the Sudeck point in an apparently healthy toddler boy: a case report. J Med Case Reports 2023;17:423. 10.1186/s13256-023-04157-9 PMC10561509 37807049

[5] Chen JC, Chen CC, Liang JT, et al. Spontaneous bowel perforation in infants and young children: a clinicopathologic analysis of pathogenesis. J Pediatr Gastroenterol Nutr 2000;30:432–5. 10.1097/00005176-200004000-00016 10776957

[6] Tan SS, Wang K, Pang W, et al. Etiology and surgical management of pediatric acute colon perforation beyond the neonatal stage. BMC Surg 2021;21:212. 10.1186/s12893-021-01213-3 33902548 PMC8077714

